# Biopsy-Based Molecular Signatures Predicting Therapeutic Response in Ulcerative Colitis

**DOI:** 10.3390/ijms27198838

**Published:** 2026-10-03

**Authors:** Ilaria Grassi, Marina Liso, Mauro Mastronardi, Anna Maria Valentini, Margherita Curlo, Raffaele Armentano, Elisabetta Cavalcanti

**Affiliations:** 1 Histopathology Unit, National Institute of Gastroenterology—IRCCS “Saverio de Bellis”, Castellana Grotte, 70013 Bari, Italy; ilaria.grassi@irccsdebellis.it (I.G.); am.valentini@irccsdebellis.it (A.M.V.); raffaele.armentano@irccsdebellis.it (R.A.); 2 IBD Unit, National Institute of Gastroenterology—IRCCS “Saverio de Bellis”, Castellana Grotte, 70013 Bari, Italy; marinaliso@libero.it (M.L.); mauro.mastronardi@irccsdebellis.it (M.M.); margherita.curlo@gmail.com (M.C.)

**Keywords:** UC, inflammation, SPARC, SPP1, IFX

## Abstract

Ulcerative colitis (UC) is a chronic inflammatory bowel disease with marked molecular heterogeneity, resulting in highly variable responses to biologic therapies. Despite the efficacy of Infliximab (IFX), many patients fail to achieve sustained remission. Identifying biomarkers predictive of treatment response is essential for advancing precision medicine in UC. Gene expression profiling of colonic mucosal biopsies from IFX responders, IFX non-responders, and healthy controls was performed using a multiplex array, followed by technical confirmation by RT-qPCR and immunohistochemical assessment to further characterize tissue expression and biological relevance. IFX non-responders displayed a stromal–immune molecular signature marked by extracellular matrix remodeling, stromal remodeling, integrin-mediated signaling, and persistent inflammatory pathways. ***SPARC*** and ***SPP1*** emerged as the most prominent biomarkers, with significantly increased gene and protein expression in non-responder patients, highlighting their association with chronic tissue remodeling and therapeutic resistance. Mucosal resistance to IFX is associated with a specific stromal–immune profile characterized by *SPARC* and *SPP1* upregulation. These findings suggest that both molecules may serve as promising candidate markers for patient stratification, highlighting the need for future prospective studies to explore their role in personalized management strategies for ulcerative colitis.

## 1. Introduction

Ulcerative colitis (UC) is a chronic, relapsing, and immune-mediated inflammatory bowel disease (IBD) characterized by continuous inflammation of the colonic mucosa. Its pathogenesis is complex and multifactorial, involving the interaction between genetic susceptibility, environmental factors, gut microbiota alterations, and dysregulated innate and adaptive immune responses [1,2].

Despite substantial advances in understanding disease mechanisms and expanding therapeutic options, UC remains a lifelong condition without a definitive cure nor a diagnostic gold-standard test, and the identification of patients at risk of an unfavorable disease course or treatment failure remains a major clinical challenge. To date, there is an urgent need for more accurate diagnostic tools and more effective therapeutic strategies. The therapeutic goals in UC have progressively shifted from symptom control toward the achievement of deeper and more objective outcomes, including corticosteroid-free clinical remission, endoscopic healing, and long-term prevention of disease progression and complications [3].

Among available advanced therapies, tumor necrosis factor (TNF)-α inhibitors have represented a major milestone in the management of moderate-to-severe UC. Infliximab (IFX), the first monoclonal antibody targeting TNF-α approved for UC, has demonstrated efficacy in inducing and maintaining clinical remission and promoting mucosal healing [4].

However, a substantial proportion of patients exhibit primary non-response or lose response over time, limiting the effectiveness of this therapeutic approach. Primary non-response to anti-TNF therapy occurs in approximately 10–40% of patients with IBD and represents a significant unmet clinical need, often requiring treatment optimization or therapeutic switching [5,6,7]. The mechanisms underlying IFX failure are complex and likely reflect the biological heterogeneity of UC. Although pharmacokinetic factors, including accelerated drug clearance and low serum drug concentrations, contribute to secondary loss of response, primary non-response may be driven by intrinsic differences in inflammatory pathways, immune cell composition, and molecular characteristics of the intestinal mucosa [8]. However, despite advances in pharmacological therapies and innovative treatment strategies, the proportion of patients achieving true disease modification remains limited. Identifying, prior to treatment initiation, those patients who are most likely to respond to a specific therapy or to an optimal treatment sequence or combination could substantially improve clinical management and patient outcomes [9,10].

Several molecular approaches, including gene profiling of bowel biopsies, have identified gene expression patterns associated with disease activity, inflammatory pathways, and response to biologic therapies [11,12]. Despite extensive research, reliable biomarkers for predicting response to anti-TNF therapy in UC are still lacking. Identifying molecular signatures capable of distinguishing responders from non-responders before treatment initiation could improve patient stratification and optimize personalized therapeutic strategies [13]. To address this unmet clinical need, this study performed gene expression profiling of colonic mucosal biopsies from UC patients classified as IFX responders (R), IFX non-responders (NR), and healthy controls (HC). Through comparative gene expression analyses, we aimed to identify molecular signatures and dysregulated pathways associated with resistance to IFX therapy. A better understanding of these mechanisms may provide novel insights into therapeutic response prediction and support the development of individualized treatment strategies in UC.

## 2. Results

### 2.1. Baseline Characteristics

Thirty (30) patients with UC and 10 control subjects were enrolled between 2022 and 2024 at the IBD Unit of the National Institute of Gastroenterology “Saverio De Bellis”, IRCCS (Castellana Grotte, BA, Italy). All UC patients had received IFX therapy and were subsequently classified as IFX-R or IFX-NR according to clinical and endoscopic outcomes. Clinical features of enrolled patients are shown in Table 1. The average age was 49.17 (range 21–80), while the average age at diagnosis was 39.73 (range 15–65). Of the total, 20 were male and 10 were female. Of the 30 patients enrolled, 20 were identified as the IFX-NR group and 10 as the IFX-R group. Other 10 patients were used as HC (features are shown in Table 1). The 100% of the IFX-NR group was classified as having severe disease (Mayo endoscopic subscore = 3). Cohort was assessment based on extent of disease: two (10%) cases were classified as E2, and eighteen (90%) cases were classified as E3, indicative of moderate and severe pathology respectively. Extraintestinal manifestations were present only in 3/20 patients. Both groups were classified according to the therapies performed: in the NR-group, 15 (75%) patients were subjected to steroid therapy. In the R-group, eight patients were classified as having moderate disease (Mayo endoscopic subscore = 2), and two patients as having severe disease (Mayo endoscopic subscore = 3). According to extent of disease, patients were classified as follows: one (10%) case as E1, four (40%) cases were classified as E2, five (50%) cases were as E3, indicative of mild, moderate and severe pathology respectively, and two (20%) patients were subjected to steroid therapy; all patients (100%) were receiving IFX therapy (Table 1).

### 2.2. Molecular Signatures of Non-Responder UC Patients

To investigate molecular mechanisms associated with IFX response in UC, we evaluated the relative mRNA expression of Epithelial–Mesenchymal Transition- (EMT), stromal-, and inflammation-related genes. RNA from the colon biopsy of HC, IFX R-UC patients and IFX NR-UC patients was analyzed by q-PCR to measure the expression of 89 selected genes specific for EMT. Overall, NR patients displayed a distinct transcriptional profile characterized by increased expression of genes involved in extracellular matrix (ECM) remodeling, integrin signaling, epithelial plasticity, and inflammatory activation. Among the analyzed genes (Figure 1), *MMP2*, *SPP1*, and *TMEM132A* exhibited the most significant increases in NR-UC patients. In particular, *MMP2* expression was significantly higher in NR-UC patients compared with both HC (*** *p* < 0.001) and R-UC patients (### *p* < 0.001), while *SPP1* showed a very strong upregulation with high statistical significance (mean difference = 28.330, 95% CI 9.975–66.630, *p* = 0.0050) (Table S2). Similarly, *TMEM132A* expression was markedly elevated in NR patients, reaching significance versus HC (*** *p* < 0.001) and responders (## *p* < 0.01). *SPARC* expression was also significantly increased in NR patients compared with both HC and responder groups (mean difference = 4.266, 95% CI 0.4577–8.989, *p* = 0.00097), further supporting its association with the non-responder phenotype (Table S2). *CAMK2N1* and *COL3A1* showed moderate but significant increases (* *p* < 0.05 vs. HC), whereas genes such as *ITGAV*, *NODAL*, *PTK2*, *AKT1*, *CALD1*, *COL1A2*, *RAC1*, and *ITGB1* displayed an upward trend in NR patients without reaching the same degree of statistical significance.

In contrast, *TIMP1*, *CTNNB1*, *KRT19*, and *IGFBP4* exhibited only limited variation among groups, suggesting a less prominent involvement in the differential molecular profile associated with treatment non-response. Overall, these findings highlight a distinct transcriptional signature in NR patients characterized by enhanced inflammatory and matrix-remodeling activity.

Based on the EMT gene expression array results, SPARC and SPP1 were selected for technical validation because they showed marked differential expression between IFX-NR and IFX-R patients and were considered biologically relevant to EMT remodeling, tissue repair, and inflammatory responses in ulcerative colitis. Therefore, SPARC and SPP1 were selected for technical confirmation by single-probe quantitative real-time PCR (RT-qPCR) in the same cohort. RT-qPCR confirmed the direction of differential expression observed in the multiplex array, with higher SPARC and SPP1 expression in IFX-NR patients compared with IFX-R and HC groups (Figure 2).

### 2.3. SPARC and SPP1 Protein Expression in Non-Responder UC Patients

To evaluate whether this transcriptional increase correlated with translational changes, protein expression was assessed via immunohistochemistry (IHC). *SPARC* expression was assessed in tissue samples from the three study groups. *SPARC* expression was predominantly localized within the stromal and inflammatory cell compartments and differed significantly among the study groups. Based on the intensity of *SPARC* immunostaining, we developed a scoring system ranging from 0 to 3+ (absent to strong) (Figure 3). NR-UC patients exhibited predominantly higher expression levels, with 35% showing moderate and 55% strong staining. In contrast, R–UC patients demonstrated overall lower *SPARC* expression, with a predominance of absent or weak staining. HC showed no detectable *SPARC* expression (100% negative) (Table 2). Statistical analysis revealed a highly significant association between NR-UC patients and *SPARC* expression (χ^2^ test, *p* < 0.001), suggesting the predictive value of this molecular axis at both the mRNA and tissue protein levels.

Subsequently, *SPP1* tissue expression was assessed in the same cohort of patients and HC. *SPP1* expression was evaluated using a scoring system ranging from 0 to 3+ (absent to strong staining) (Figure 4). *SPP1* expression scores differed significantly between NR-UC, R-UC and HC, with higher scores observed in the NR-UC group. In NR-UC patients, *SPP1* was predominantly expressed at intermediate to high levels, with 45% of cases showing moderate (2+) and 40% strong (3+) staining, while only 15% exhibited weak expression and no cases showed complete absence. In contrast, patients’ R-UC demonstrated substantially lower expression, with 80% of samples negative for *SPP1*, 20% showing weak expression, and no cases displaying moderate or strong staining. HC consistently showed no detectable *SPP1* expression (100% absent staining) (Table 3). Overall, *SPP1* intensity scores differed significantly across groups, with higher expression in inflammatory and stromal component associated with progression disease and a progressive reduction observed in remission and healthy conditions. Statistical analysis confirmed a highly significant association between patient NR-UC and *SPP1* expression (χ^2^ test, *p* < 0.001), suggesting that *SPP1* upregulation is associated with persistent inflammatory activity and may represent a marker of disease severity and poor therapeutic response in UC.

## 3. Discussion

In this study, we identified a transcriptional signature associated with non-response to IFX therapy in patients with UC. Comparative analysis between R-UC and NR-UC patients identified a set of 18 genes selected based on differential expression patterns and their potential biological relevance to UC, including *SPARC*, *SPP1*, *ITGB1*, *COL3A1*, *CTNNB1*, *TMEM132A*, *COL1A2*, *CALD1*, *MMP2*, *ITGAV*, *PTK2*, *NODAL*, *TIMP1*, *IGFBP4*, *KRT19*, *AKT1*, *CAMK2N1* and *RAC1*. Functional interpretation of these genes suggests that IFX resistance is associated with activation of stromal remodeling, ECM organization, integrin-mediated signaling, and survival pathways rather than with a purely TNF-driven inflammatory profile [14,15,16]. In IBD, the composition and functional properties of intestinal fibroblasts and myofibroblasts are profoundly altered [17]. These mesenchymal stromal cells acquire an activated phenotype characterized by increased production of pro-inflammatory cytokines and ECM components, contributing to tissue remodeling and fibrosis [18]. Several studies have shown that IBD-associated fibroblasts play a key role in anti-TNF therapy refractoriness, highlighting the difficulty of directly targeting these cell populations in clinical settings [17,19,20]. This suggests that a deeper understanding of the cellular and molecular mechanisms governing the EMT cells from a healthy to an inflammatory or fibrosis-associated state is required, as it may help elucidate the complex pathogenesis of both UC and Crohn’s disease and identify novel therapeutic targets for intestinal fibrosis. When patients develop resistance to anti-TNF therapy, inflammation is no longer purely immune-driven but becomes strongly associated with structural tissue remodeling. This is characterized by progressive fibrosis, leading to tissue thickening and possible strictures, along with disruption of normal mucosal architecture due to excessive extracellular matrix deposition. In parallel, a persistent wound-healing response is maintained, reflecting chronic, unresolved tissue injury and ongoing remodeling processes. In this study we identified several genes involved in ECM deposition and tissue remodeling, including *COL1A2*, *COL3A1*, *MMP2*, *TIMP1*, *SPP1* and *SPARC*. Persistent activation of these pathways may reflect chronic mucosal damage and fibroblast/myofibroblast activation in NR patients. Previous studies have shown that anti-TNF resistance is frequently associated with a shift from acute inflammatory pathways toward mesenchymal and stromal programs characterized by fibrosis, altered tissue architecture, and sustained wound-healing responses [21]. In particular, *SPARC* and *MMP2* have been implicated in ECM remodeling and chronic intestinal inflammation, whereas increased *TIMP1* expression has been associated with altered ECM turnover and persistent mucosal inflammation in UC [22,23]. A second major functional cluster identified in NR patients involves integrin signaling and cell survival pathways, including *ITGA5*, *ITGB1*, *PTK2 FAK*, *AKT1*, and *RAC1*. Integrin-mediated signaling regulates leukocyte trafficking, epithelial cell adhesion, and immune–stromal cell interactions, thereby contributing to intestinal immune homeostasis and chronic inflammation in ulcerative colitis [24]. Moreover, activation of the PI3K/AKT and JAK/STAT signaling pathways promotes immune cell survival and resistance to apoptosis, potentially sustaining inflammatory pathways despite TNF blockade and contributing to reduced therapeutic responsiveness [25,26]. Notably, integrin signaling has emerged as a critical pathway in IBD, as demonstrated by the clinical efficacy of anti-integrin therapies such as VDZ [27]. Our findings therefore support the hypothesis that NR patients may rely on alternative inflammatory circuits independent of TNF signaling. *SPARC* is a matricellular protein that regulates ECM remodeling and collagen fibrogenesis. Overexpression of the *SPARC* gene suggests that persistent intestinal fibrosis and impaired epithelial barrier function may be key contributors to treatment resistance. Recent studies have shown that elevated *SPARC* levels contribute to refractoriness in chronic IBD through multiple mechanisms, including disruption of the intestinal barrier, increased collagen deposition, and immunometabolic alterations [12,28]. In particular, high *SPARC* expression has been reported to promote the polarization of anti-inflammatory macrophages toward a pro-inflammatory phenotype, thereby sustaining localized inflammation that is resistant to treatment [29]. The identification of SPARC overexpression prior to therapy may warrant further investigation as a potential biomarker of treatment response in IBD. Patients with high *SPARC* expression or a fibrotic molecular profile are less likely to achieve mucosal healing in response to anti-TNF therapy. In our cohort, the significant upregulation of *SPARC* supports its role as a potential driver of mucosal structural remodeling, which may impair therapeutic response by sustaining a pro-fibrotic tissue microenvironment. In parallel, *SPP1* (*Osteopontin*) was significantly overexpressed in NR patients. *SPP1* is a multifunctional cytokine-like protein involved in macrophage activation, Th1/Th17 immune responses, and chronic intestinal inflammation [30,31]. Recent biomarker studies in IBD have identified *Osteopontin* as a central mediator of persistent immune activation and chronic tissue inflammation, particularly in biologic-refractory disease where alternative inflammatory pathways sustain disease activity despite TNF blockade [32]. In our cohort, the strong association between *SPP1* expression and non-responder status further supports its role as both a biomarker and a potential contributor to anti-TNF resistance. The concomitant overexpression of *SPARC* and *SPP1* suggests a coordinated stromal–immune axis underlying IFX non-response. This dual signature integrates ECM remodeling (*SPARC*-driven) with sustained inflammatory signaling (*SPP1*-driven), defining a biologically distinct UC profile characterized by both tissue restructuring and immune persistence. Importantly, this aligns with recent multi-omics evidence from large IBD cohorts which has demonstrated that integrative molecular profiling can identify patient subgroups with distinct inflammatory and structural trajectories and differential response to biologic therapies [12]. We acknowledge that baseline disease severity (Mayo endoscopic subscore) and extent were higher in the NR-UC group. This imbalance implies that *SPARC* and *SPP1* upregulation might partially reflect a greater inflammatory burden and advanced tissue remodeling. Given our sample size, robust multivariate adjustment was limited, highlighting the need for validation in larger, propensity score-matched cohorts. From a translational perspective, SPARC and SPP1 may represent potential candidate biomarkers associated with IFX response, although their predictive value requires confirmation in larger and independently validated cohorts.

Their combined assessment could improve patient stratification prior to anti-TNF therapy initiation, a critical unmet need in UC management given that up to 30–40% of patients fail to achieve sustained remission with IFX. More broadly, these findings support a shift from a cytokine-centered model of UC toward a multidimensional framework that incorporates stromal remodeling, immune activation, and tissue architecture as determinants of therapeutic response. Together, the *SPARC/SPP1* stromal–immune axis correlates strongly with primary anti-TNF failure in UC. These findings identify SPARC and SPP1 as potential candidate molecular markers associated with IFX non-response and provide a rationale for further investigation in larger, prospective, and independently validated cohorts.

## 4. Materials and Methods

### 4.1. Ethical Considerations

The study protocols on human subjects were conducted in accordance with the principles of the Declaration of Helsinki (1964) and approved by the local Ethics Committee, “Comitato Etico I.R.C.C.S.-Istituto Tumori “Giovanni Paolo II”-Bari” (protocol code Number 506 on 11 October 2022).

### 4.2. Study Population

In total, 30 patients with ulcerative colitis and 10 control subjects were enrolled between 2022 and 2024 at the IBD Unit of the National Institute of Gastroenterology “Saverio De Bellis”, IRCCS (Castellana Grotte, BA, Italy) and underwent IFX infusion. All enrolled patients with ulcerative colitis (UC) received a standard Infliximab (IFX) induction regimen consisting of intravenous infusions of 5 mg/kg at weeks 0, 2, and 6, followed by maintenance infusions every 8 weeks. Colonic mucosal biopsies were collected from all UC patients at baseline, prior to the administration of the first Infliximab infusion.

In accordance with our institutional protocol, anti-Infliximab antibody (anti-IFX Ab) levels were routinely monitored at the 3rd, 5th, and 8th infusions.

These pharmacokinetic and pharmacological assessments were not used as standalone criteria for defining primary IFX non-response. Treatment response was assessed at week 14 using the Mayo Clinic Score (MCS) for clinical disease activity and the Mayo Endoscopic Score (MES) for endoscopic disease activity, in accordance with established ECCO recommendations [32,33]. Clinical response was defined according to the Mayo-based clinical response criteria, while endoscopic response was assessed using the MES. Patients who fulfilled both the predefined clinical and endoscopic response criteria at week 14 were classified as IFX responders (R), whereas patients who did not fulfill both criteria were classified as primary IFX non-responders (NR). Accordingly, IFX trough concentrations, including values below 2 µg/mL, were considered pharmacokinetic indicators of potentially inadequate drug exposure and were not, by themselves, sufficient to classify a patient as a primary non-responder. Patients experiencing secondary loss of response during the maintenance phase were excluded from this study.

The diagnoses of IBD were based on standard clinical, radiological, endoscopic, and histological criteria [34,35]. For classification of UC patients, the Montreal classification [36,37] was used. Anatomic location was based on the Montreal classification, based on the criteria ulcerative proctitis (E1), left-sided UC (distal UC; E2), and extensive UC (pancolitis; E3). Patients with indeterminate colitis were excluded from the study. The clinical characteristics of the IBD study population are shown in Table 1.

### 4.3. RNA Extraction

Total RNA was isolated from formalin-fixed, paraffin-embedded (FFPE) of 10 μm thickness using RNeasy FFPE kit (73504, Qiagen, Hilden, Germany) according to the manufacturer’s protocol including the treatment of sections with Deparaffinization Solution (19093Qiagen, Hilden, Germany). Total RNA was then eluted in 30 μL of ribonuclease-free water. The RNA concentration was determined with the NanoDrop ND-2000 Spectrophotometer (Nanodrop Technologies, Wilmington, DE, USA).

### 4.4. Quantitative Polymerase Chain Reaction Analysis

Total RNA (1 µg) was reverse transcribed using an iScript complementary DNA Synthesis kit (Bio-Rad, Hercules, CA, USA) with random primers for complementary DNA synthesis. Gene expression of 89 genes was performed using the Epithelial–Mesenchymal Transition (EMT) (SAB Target List) H96 (Bio-Rad, Hercules, CA, USA). Real-time analysis was performed on a CFX96 Touch System (Bio-Rad), and for the relative expression the Delta Delta Threshold Cycle (ΔΔCt) method was used. Gene cluster analyses were performed with CFX Manager software 3.1 (Bio-Rad, Hercules, CA, USA).

### 4.5. Immunohistochemistry

Immunohistochemistry (IHC) analyses for Anti-*SPARC* antibody (clone SP205, Abcam, Cambridge Biomedical Campus, UK) and Anti-*Osteopontin* antibody (clone 7C5H12, Abcam, Cambridge Biomedical Campus, UK) were performed in the FFPE tissues obtained from UC patients and controls. Distal colon sections (4 mm) were freshly cut, mounted on Apex Bond Slides (3800040, Leica Biosystems, Deer Park, IL, USA) and dried at 60 °C for 30 min. IHC analysis was performed on a Dako Omnis 165 Duo automated immunostainer (Agilent Technologies, Zürich, Switzerland), from deparaffinization to counterstaining with hematoxylin, using the EnVision FLEX High pH Kit (GV823, Dako Omnis, Agilent Technologies, Zürich, Switzerland). A dilution of 1:200 was used for the evaluation of anti-*SPARC*, while for that of Anti-*Osteopontin*, 1:50 was used. Antigen retrieval was performed using Target Retrieval Solution, High pH (50x Tris/EDTA buffer, pH 9) (K8004, Dako Omnis, Agilent Technologies, Santa Clara, CA USA). Negative control sections were evaluated using the above methodology. Anti-*SPARC* and Anti-*Osteopontin* expression were scored for all staining patterns, according to both the staining intensity and the percentage of positively stained cells, by two independent, blinded pathologists; discrepancies in the interpretation of scoring were resolved by consensus. Anti-*SPARC* and Anti-*Osteopontin* expression were assessed using a semi-quantitative categorical scoring system (0 to 3+) based on the percentage of positive cells in the inflammatory and stromal components and the intensity of immunostaining. Specifically, Anti-*SPARC* and Anti-*Osteopontin* expression were assessed as follows: 0 (no staining) negative; 1, weak expression, staining in <1% of the inflammatory and stromal components; 2, moderate expression ≥5% of the inflammatory and stromal components; and 3, strong staining in ≥10% of the inflammatory and stromal components (Figure 3 and Figure 4). Images were acquired using the NanoZoomer S20 Digital Slide Scanner (Hamamatsu Photonics, Arese, Italy) at 20× magnification.

### 4.6. Statistical Analysis

Statistical analyses were performed using GraphPad Prism software, version 5.0 for Windows (GraphPad Software, San Diego, CA, USA). The individual patient was considered the experimental unit for all quantitative analyses. Each patient-derived colonic biopsy represented one biological replicate. For quantitative PCR and RT-qPCR analyses, each sample was analyzed in technical triplicate to ensure analytical precision. The mean value of the technical replicates was calculated for each patient and used as a single observation in the subsequent statistical analyses. Technical replicates were therefore not considered independent biological observations. Quantitative data are presented as mean ± standard error of the mean (SEM), unless otherwise indicated. For comparisons between two groups, a two-tailed Student’s *t*-test was used. For comparisons among multiple groups, one-way analysis of variance (ANOVA) followed by Bonferroni’s post hoc test was applied, as appropriate. Results were considered statistically significant when *p* < 0.05. For the multi-gene qPCR array screening of 89 genes, the Benjamini–Hochberg false discovery rate (FDR) procedure was applied to correct for multiple hypothesis testing. Genes were considered significantly differentially expressed when the adjusted *p*-value was <0.05. For the downstream single-gene validation experiments, statistical significance was assessed using the appropriate two-group or multiple-group comparison test, as described above, and a nominal *p*-value < 0.05 was considered statistically significant. Exact *p*-values and, where applicable, confidence intervals are reported in the manuscript and/or figure legends to ensure transparency of the statistical analyses.

## Figures and Tables

**Figure 1:**
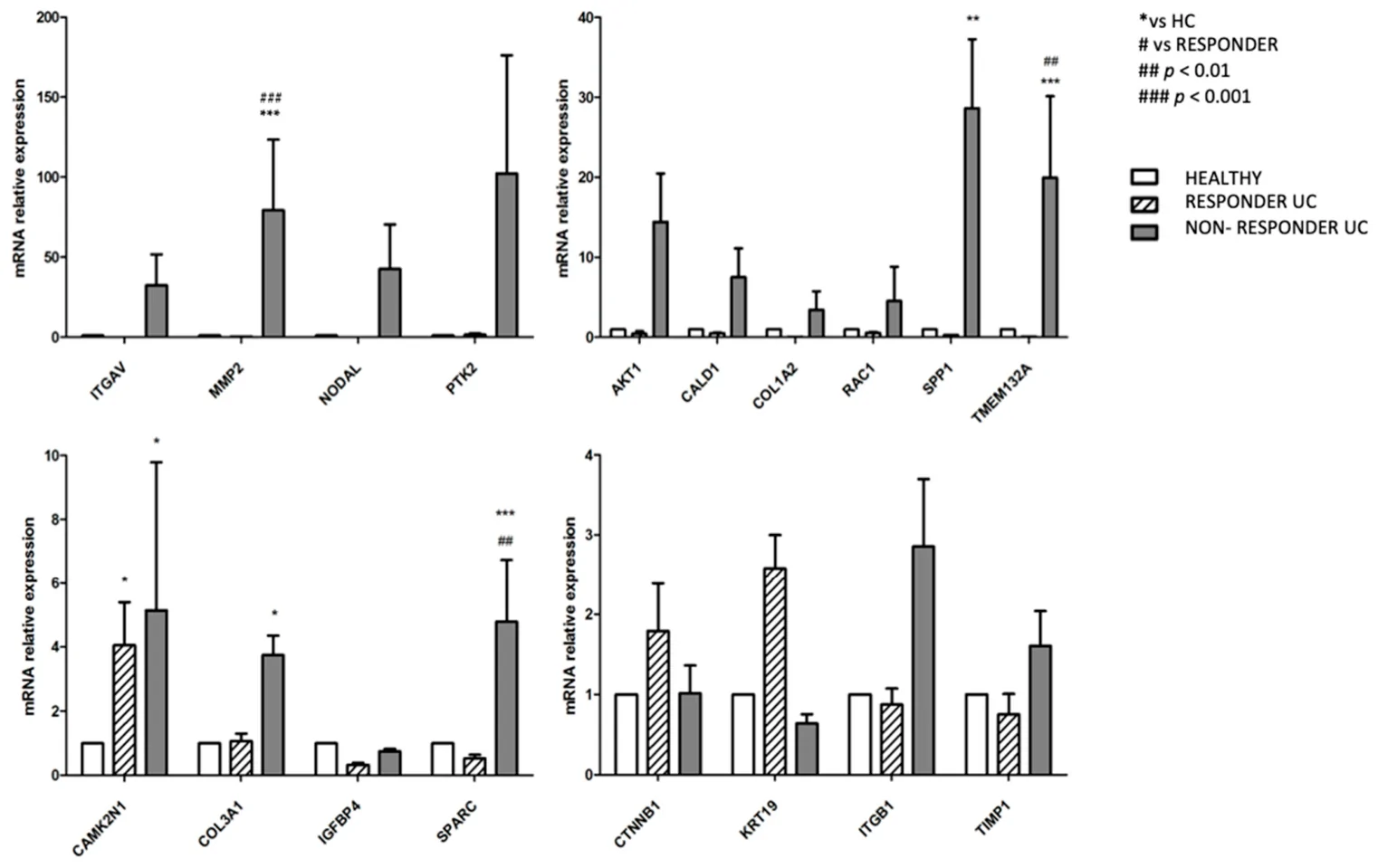
**Expression analysis of EMT-related genes in UC responders, non-responders, and healthy controls.** Data are presented as mean ± SEM of individual patient samples within each clinical group. Each patient-derived sample represents one biological replicate. For each sample, qPCR measurements were performed in technical triplicate, and the mean of the technical replicates was used for statistical analysis. Technical replicates were not considered independent observations (* *p* < 0.05, ** *p* < 0.01, *** *p* < 0.001). For the 89-gene screening, statistical significance was assessed after Benjamini–Hochberg FDR correction, with Padj < 0.05 considered significant. Nominal and adjusted *p*-values for the selected genes are reported in Supplementary Table S1.

**Figure 2:**
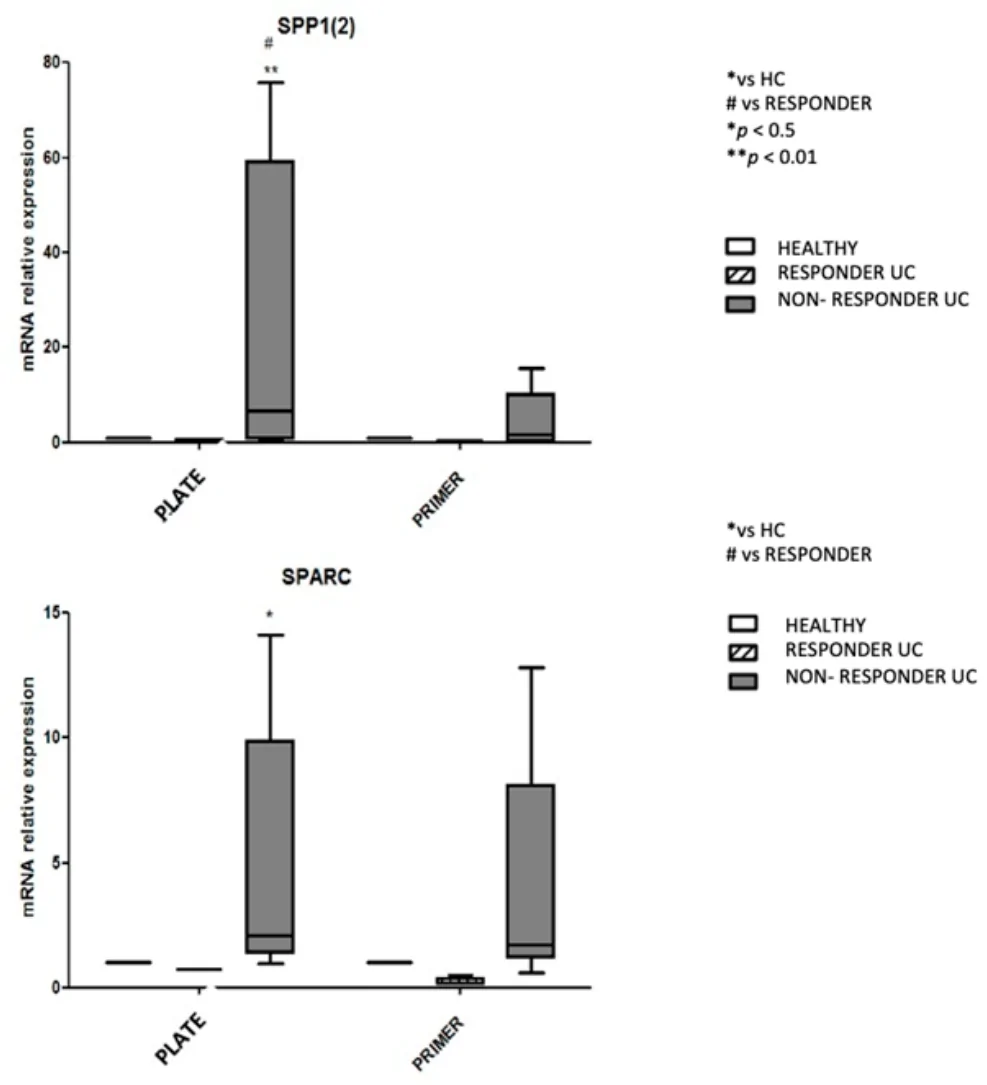
RT-qPCR confirmation of SPARC and SPP1 differential expression in IFX-NR patients compared with HC and R-UC groups. (# *p* < 0.05).

**Figure 3:**
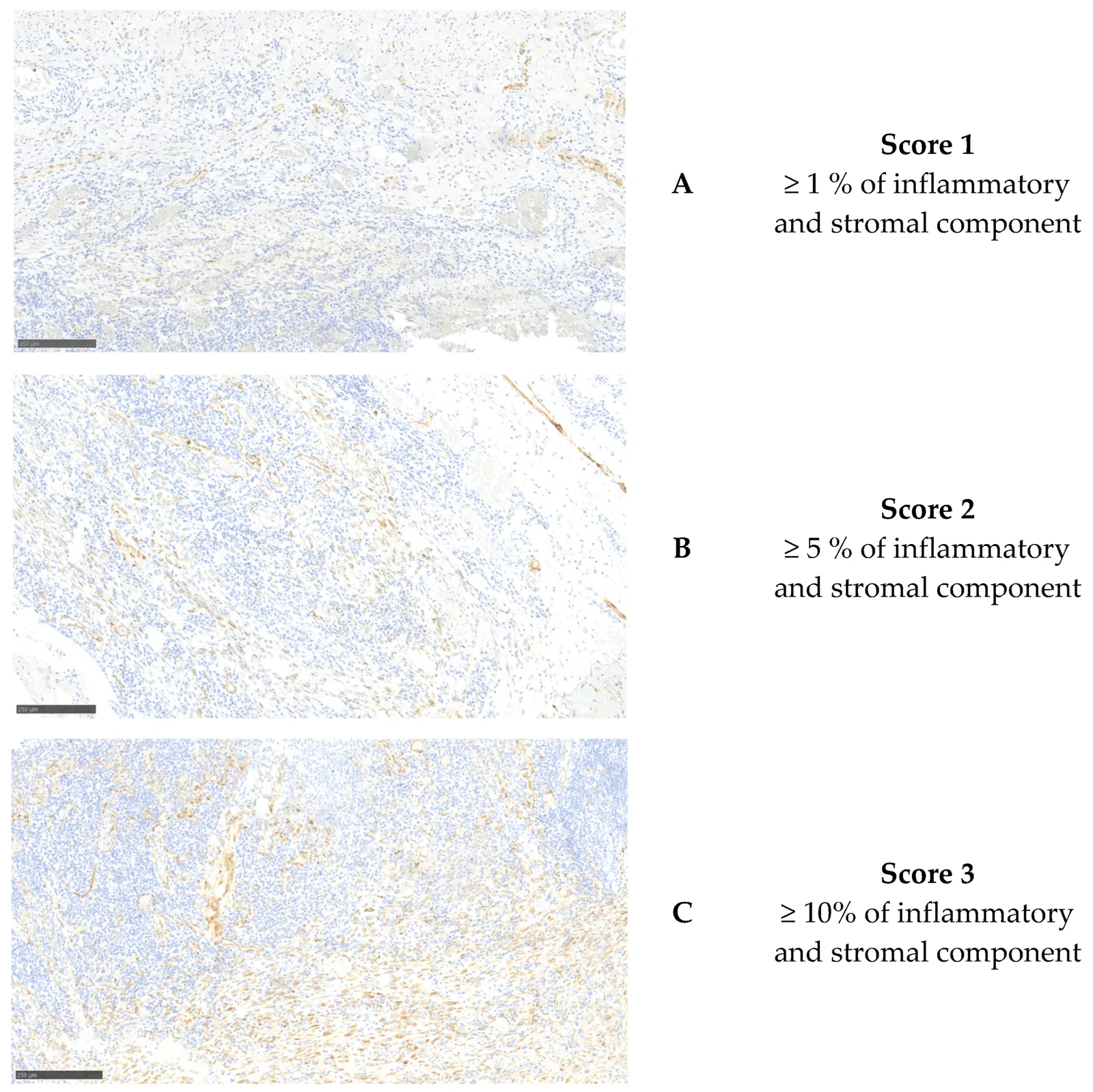
Representative patterns of *SPARC* staining intensity: (**A**) weak expression (**B**) medium expression; (**C**) strong expression (magnification ×20).

**Figure 4:**
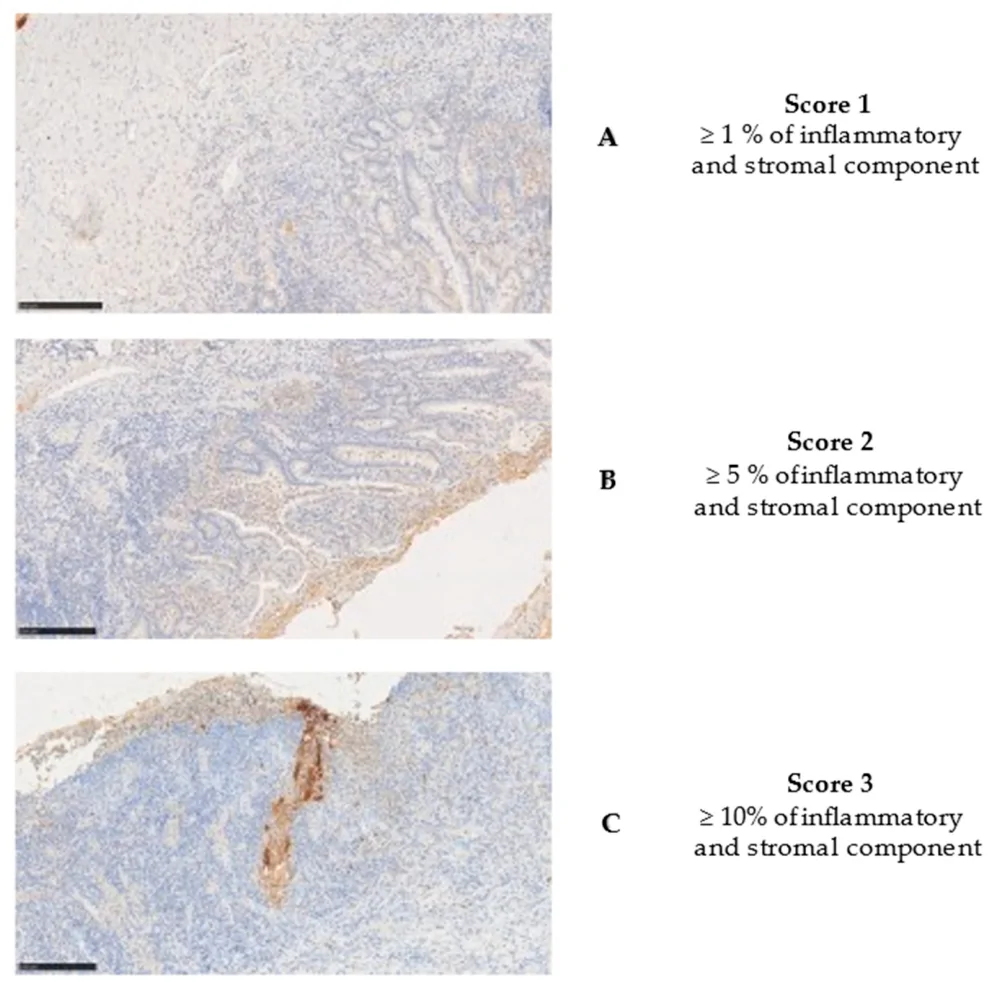
Representative patterns of *SPP1* staining intensity: (**A**) weak expression (**B**) medium expression; (**C**) strong expression (magnification ×20).

**Table 1:** Clinicopathologic features of enrolled patients.

		UC Non-Responder (20)		UC Responder (10)		Control (10)	
		**n**	**%**	**n**	**%**	**n**	**%**
**Gender**	Male	14	70	6	60	7	70
	Female	6	30	4	40	3	30
**Age** (Median, range)		53.25 (23–80)		45.1 (21–70)		48.3 (21–75)	
**Age at diagnosis** (Median, range)		45.35 (15–65)		34.12 (16–45)			
**Mayo Score**	0–1						
	2			8			
	3	20	100	2			
**Extent of Disease**	E1			1	10		
	E2	2	10	4	40		
	E3	18	90	5	50		
**Extraintestinal Manifestations**	Present	3					
	Absent	17					
**Steroid Therapy**	Yes	15	75	2	20		
	No	5	25	8	80		

E1—proctitis (proximal extent to the sigmoid colon). E2—Left-sided (to the splenic flexure). E3: Pancolitis (beyond the splenic flexure).

**Table 2:** *SPARC* intensity scores in the inflammatory and stromal component.

SPARC Expression					
	Absent (0)	Weak (1+)	Medium (2+)	Strong (3+)	
	n (%)	n (%)	n (%)	n (%)	*p*-Value *
**UC patients NR**		2 (10)	7 (35)	11 (55)	***p* < 0.001**
**UC patients R**	7 (70)	2 (20)	1 (10)		
**Healthy Control**	10 (100)				

The *p*-value refers to the statistical comparison of SPARC expression among the study groups. A *p*-value < 0.05 was considered statistically significant. * *p* < 0.001.

**Table 3:** *SPP1* intensity scores in the inflammatory component.

SPP1 Expression					
	Absent (0)	Weak (1+)	Medium (2+)	Strong (3+)	
	n (%)	n (%)	n (%)	n (%)	*p*-Value *
**NR-UC patients**		3 (15)	9 (45)	8 (40)	*p* < 0.001
**R-UC patients**	8(80)	2(20)			
**Healthy Control**	10 (100)				

The *p*-value refers to the statistical comparison of SPP1 expression among the study groups. A *p*-value < 0.05 was considered statistically significant. * *p* < 0.001.

## Data Availability

The original contributions presented in this study are included in the article/Supplementary Materials. Further inquiries can be directed to the corresponding author.

## Supplementary Materials

The following supporting information can be downloaded at: https://www.mdpi.com/article/10.3390/ijms27198838/s1.

## Abbreviations

The following abbreviations are used in this manuscript:

UC	ulcerative colitis
IBD	inflammatory bowel disease
IFX	Infliximab
TNF-a	tumor necrosis factor-alpha
EMT	Epithelial–Mesenchymal Transition
ECM	extracellular matrix
R-UC	ulcerative colitis responder
NR-UC	ulcerative colitis non-responder
HC	healthy control
SPARC	Secreted Protein Acidic and Rich in Cysteine
SPP1	Secreted Phosphoprotein 1
RT-qPCR	real-time quantitative PCR
FFPE	formalin-fixed, paraffin-embedded
IHC	immunohistochemistry
