# Supplementary material for: Biopsy-Based Molecular Signatures Predicting Therapeutic Response in Ulcerative Colitis
Source: Int J Mol Sci. 2026 Oct 3;27(19):8838. doi: 10.3390/ijms27198838 (PMC13664742; doi:10.3390/ijms27198838)
Supplement: Supplementary file 1 [file ijms-27-08838-s001.zip › ijms-4474901-supplementary.pdf]

Supplementary Table S1. Gene expression analysis of the 89-gene EMT-related qPCR array.

ID Gene	Fold Change*	HC vs NR Nominal p-value	FDR-adjusted p-value)	NR vs R Nominal p-value	FDR-adjusted p-value
**MMP2**	2.86	0.00044	0.00396	0.00038	0.00342
**SPP1**	28.64	0.00071	0.00426	0.0050	0.00550
**TMEM132A**	19.97	0.00098	0.01320	0.0010	0.02664
**SPARC**	4.79	0.00031	0.00396	0.00097	0.00126
**AKT1**	14,46	0.0176	0.06336	0.0426	0.10954
**PTK2**	102.2	0.0229	0.06870	0.0285	0.08550
**CAMK2N1**	5.14	0.0307	0.04894	0.0361	0.0416
**COL3A1**	3.74	0.0352	0.04270	0.0478	0.03005
**ITGB1**	9.2	0.0762	0.15240	0.0834	0.16362
**TIMP1**	1.61	0.283	0.48109	0.155	0.25364
**CALD1**	7.52	0.294	0.48109	0.259	0.38850
**NODAL**	2.70	0.476	0.66738	0.588	0.68160
**KRT19**	0.64	0.482	0.66738	0.379	0.487
**ITGAV**	32.42	0.532	0.68400	0.649	0.73013
**RAC1**	4.56	0.634	0.76080	0.568	0.68160
**COL1A2**	3.44	0.727	0.81788	0.702	0.74329
**IGFBP4**	0.75	0.874	0.92541	0.808	0.80800
**CTNNB1**	1.79	0.975	0.97500	0.0909	0.16362

*Fold change shown here is the mean expression in IFX-NR relative to HC.
Nominal and FDR-adjusted p-values are reported for comparisons between HC and IFX-NR and between IFX-NR and IFX-R.

Supplementary Table S2. Effect estimates and 95% confidence intervals and nominal p-value SPARC and SPP1 expression.

Gene	Comparison	Effect estimate (mean difference)	95% CI	p-value
SPARC	IFX-NR vs HC	3.786	0.127–7.444	0.00031
SPARC	IFX-NR vs IFX-R	4.266	0.4577–8.989	0.00097
SPP1	IFX-NR vs HC	27.640	3.412–51.860	0.00071
SPP1	IFX-NR vs IFX-R	28.330	9.975–66.630	0.005
